# Mesenchymal stromal cells for *Pseudomonas aeruginosa* pneumonia: mechanisms, preclinical evidence, and translational barriers

**DOI:** 10.3389/fmicb.2025.1674456

**Published:** 2025-10-20

**Authors:** Zhuo-ling Zhang, Ze Yu, Yue-chuan Shen

**Affiliations:** ^1^Department of Emergency, Zhoushan Hospital, Wenzhou Medical University, Zhoushan, China; ^2^Laboratory of Cytobiology and Molecular Biology, Zhoushan Hospital, Wenzhou Medical University, Zhoushan, China

**Keywords:** mesenchymal stromal cells, *Pseudomonas aeruginosa*, pulmonary infection, immuneregulation, therapeutic progress

## Abstract

*Pseudomonas aeruginosa*, as an important pathogenic bacterium in nature, is particularly prone to cause severe pulmonary infections in patients with weakened immune function, which poses a significant threat to the survival and quality of life of patients. In recent years, Mesenchymal stromal Cells (MSCs) have become a hot topic in the antibacterial research due to their superior immunomodulatory ability and tissue repair characteristics. Existing studies have shown that MSCs have significant potential in improving the immune response to pulmonary infections and promoting tissue repair and regeneration. However, although multiple studies have explored the application of MSCs in pulmonary infections, there are still problems such as unclear mechanisms and high difficulty in clinical translation. This article aims to summarize the latest research progress of MSCs in the treatment of *Pseudomonas aeruginosa* pulmonary infections, analyze their potential mechanisms of action, clinical application situations, and future research directions, and provide references for in-depth clinical and basic research in this field.

## 1 Introduction

*Pseudomonas aeruginosa* is a Gram-negative bacterium found in moist environments, posing serious infection risks to humans, especially in hospitals (Marei, 2020; de Sousa et al., 2021). The pathogenicity of *Pseudomonas aeruginosa* is closely related to its various virulence factors, including the formation of cellular biofilms, drug resistance, and multiple secreted toxins, making it particularly dangerous in patients with compromised immune systems, the host of *Pseudomonas aeruginosa* leads to vulnerability in immunosuppressed for *Pseudomonas aeruginosa* infection (Azam and Khan, 2019; Khan, 2024).

*Pseudomonas aeruginosa* is a major nosocomial pathogen, causing 8.5% of hospital-acquired infections, especially in immunocompromised patients, with 36 infections per 10,000 discharges; it is prevalent in pneumonia and burn wounds, linked to ICU techniques, immunocompromised patients, and antibiotic use (Reynolds and Kollef, 2021). In the patients with weakened immune function, *Pseudomonas aeruginosa* infection shows a higher incidence and mortality rate.

Traditional treatment methods, such as antibiotic therapy, are facing an increasingly serious problem of drug resistance. *Pseudomonas aeruginosa* shows significant resistance to a variety of antibiotics, especially to drugs such as *β*-lactam, aminoglycosides and quinolones (Pang et al., 2019; Zagui et al., 2024). Such severe drug resistance complicates clinical treatment and leads to treatment failure, increasing the risk of death for patients (de Sousa et al., 2021). Therefore, seeking new treatment strategies has become an important direction in current medical research.

MSCs have received extensive attention due to their unique biological characteristics. Studies have shown MSCs can alleviate the inflammatory response caused by infection through multiple mechanisms and promote tissue repair, thus demonstrating potential application prospects in the treatment of *Pseudomonas aeruginosa* infection (Peterson et al., 2021; Esfandiary et al., 2024; Mitra et al., 2024). With the in-depth research on MSCs, their application potential in clinical treatment is increasingly evident, especially in combating drug- resistant bacterial infections (Jiang et al., 2021; Enayati et al., 2024).

## 2 Pathophysiological mechanism of *Pseudomonas aeruginosa* pulmonary infections

### 2.1 Cellular responses during the infection process

*Pseudomonas aeruginosa* is a common opportunistic pathogen, especially prominent in chronic pulmonary infections. The infection process involves complex cellular responses, mainly including the activation of immune cells and inflammatory responses. When *Pseudomonas aeruginosa* invades the lungs, the host’s immune system triggers an immune response by recognizing the pathogen-associated molecular patterns (PAMPs) of the bacteria. Macrophages and neutrophils are the first immune cells to respond, fighting infections through phagocytosis and the release of inflammatory mediators such as cytokines and chemokines (Thomsen et al., 2021). Studies have shown that in chronic pulmonary infections, *Pseudomonas aeruginosa* can evade the host’s immune clearance through the formation of its biofilm. This biofilm not only provides a physical barrier but also weakens the effect of antibiotics, leading to treatment failure (Beg et al., 2023). In addition, presence of pathogens can cause persistent inflammatory responses, leading to damage and functional disorders of lung tissue, and thereby exacerbating patient’s condition (Li et al., 2023). Therefore, a thorough understanding of the cellular responses during the infection process is crucial for formulating effective treatment strategies.

### 2.2 The virulence factor of *Pseudomonas aeruginosa*

The virulence factors of *Pseudomonas aeruginosa* are the key to its pathogenicity, mainly including exotoxins, enzymes and biofilms. Exotoxins such as toxin A and elastase can directly damage host cells, triggering apoptosis and tissue damage (Jurado-Martín et al., 2021). Besides, *Pseudomonas aeruginosa* secretes various enzymes, such as proteases and lipases, which can break down the immune response components of the host and thereby enhance its survival ability (Rickard et al., 2024). The formation of biofilms is one of the most important adaptive mechanisms of *Pseudomonas aeruginosa* in pulmonary infections. This biofilm not only resists antibiotics but also protects the bacteria from attacks by the host’s immune system (Shoaib et al., 2019). Research has found that the biofilm of *Pseudomonas aeruginosa* contains functional amyloid proteins, which play a significant role in the stability of the biofilm and the adaptability of the bacteria (Beg et al., 2023). Therefore, research on these virulence factors provides potential targets for the development of novel anti-infective therapies.

### 2.3 Immune escape mechanism

The immune escape mechanism of *Pseudomonas aeruginosa* in the host immune system is complex and diverse. Its main strategies include altering antigen, secreting immunosuppressive factors and forming biofilms, etc. Research shows that *Pseudomonas aeruginosa* can evade recognition by the host’s immune system by regulating the expression of its surface antigens, thereby leading to the occurrence of chronic infections (Zhou et al., 2023). In addition, pseudomonas protease LasB secreted by *Pseudomonas aeruginosa* can promote the activation of IL-1β, neutrophil inflammation and the destruction of lung structure, which further inhibits the host’s immune response (Sun et al., 2020). The formation of biofilms not only provides a physical barrier but also inhibits function of immune cells by altering the local microenvironment, leading to the occurrence of immune escape (Eiselt et al., 2024). The existence of these mechanisms makes *Pseudomonas aeruginosa* exhibit extremely strong pathogenicity and drug resistance in the pulmonary infections. Therefore, research on its immune escape mechanism will provide an important basis for the development of new immunotherapies.

## 3 The biological characteristics of mesenchymal stromal cells

### 3.1 Source and classification

Mesenchymal stromal cells (MSCs) are a type of adult stem cells with self-renewal and multi- directional differentiation potential. According to their sources, MSCs can be classified into several types, including bone marrow-derived mesenchymal stem cells (BMSCs), etc. MSCs from different sources show significant differences in biological characteristics and functions. For instance, AD-MSCs have become a popular choice in regenerative medicine research due to their easy accessibility and high proliferation ability, while BMSCs have been widely applied in clinical trials because of their strong multi-directional differentiation potential (Yao et al., 2021; Sanmartin et al., 2022). In addition, in recent years, MSCs derived from induced pluripotent stem cells (iPSCs) have also attracted attention because they theoretically have the potential for unlimited expansion and can overcome the limitations of traditional tissue-derived MSCs (Zhao and Liu, 2020).

### 3.2 The mechanism of immune regulation

MSCs have demonstrated significant potential in immune regulation, mainly influencing the function of the immune system through multiple mechanisms. MSCs can secrete various cytokines and growth factors, such as transforming growth factor β (TGF-β), interleukin-10 (IL-10), etc. These factors can inhibit the activation of T cells and B cells, reduce inflammatory responses, and thereby exert immunosuppressive effects (Jovic et al., 2022; Sanmartin et al., 2022). In addition, MSCs can further influence the immune response by regulating the functions of dendritic cells, macrophages and natural killer cells. For instance, studies have shown that MSCs can promote the generation of regulatory T cells and inhibit the proliferation of effector T cells. This mechanism is of great significance in autoimmune diseases and transplant rejection reactions (Sanmartin et al., 2022; Murohara, 2020).

MSC and its derivatives can down-regulate inflammatory responses. For instance, in the corneal epithelial cell model infected with *Pseudomonas aeruginosa*, MSC-CM significantly down-regulated IL-6 and TNF-*α* expression induced by lipopolysaccharide, thereby alleviating inflammation. In addition, the antibacterial factors secreted by MSCs help directly inhibit bacterial growth. *In vitro* studies, MSC-CM significantly inhibited the growth of *Pseudomonas aeruginosa* and upregulated the expression of the antimicrobial peptide Lipocalin 2 and LL-37 (Mitra et al., 2024).

Studies have shown that MSCs can affect the phenotype of macrophages. In sonodynamic therapy, PMZMU (MSC-based nanoplatform) promotes the polarization of macrophages toward the M2 phenotype, thereby reducing inflammatory damage and improving infection outcomes. This indirectly supports immune responses regulation by MSCs via macrophage reprogramming (Sarkar et al., 2025b). In addition, MSC extracellular vesicles (MSC-EVs) are the key carriers of the anti-infective effect of MSC. In the genetic disease Cystic Fibrosis (CF) mouse model, MSC EVs reduce acute *P. aeruginosa* lung infection and inflammation, demonstrating their dual functions of antibacterial and immunomodulatory (Sarkar et al., 2025a).

## 4 Research on the application of MSCs in *Pseudomonas aeruginosa* infection

### 4.1 Research progress in animal models

In a mouse model of cystic fibrosis (CF) caused by acute *Pseudomonas aeruginosa* pulmonary infection, researchers used clinically isolated *Pseudomonas aeruginosa* strains, inoculated at a dose of approximately 5 × 10^5^ CFU, and treated with MSC-derived extracellular vesicles (MSC EVs). The results showed that MSC EVs could significantly reduce the Pseudomonas burden in the lungs, decrease the levels of pro-inflammatory cytokines (such as IL-6, TNF-*α*) and immune cell infiltration in BAL, and improve the survival rate of infected mice. Histological assessment indicates that the inflammatory damage has been alleviated, and the relevant summary table has been presented in Supplementary table (Sarkar et al., 2025a). The research results of these animal models provide strong support for effectiveness of MSC in clinical applications and lay a foundation for further research.

### 4.2 Clinical trial results and analysis

In clinical trials, the application of MSCs has gradually demonstrated its potential in treating *Pseudomonas aeruginosa* infections. Although the number of specialized clinical trials targeting this bacterium is currently limited, existing studies on non-*Pseudomonas aeruginosa* infections have shown that MSC treatment has positive effects in improving patients’ lung function and reducing infection-related complications. For instance, research has found that MSCS can improve the clinical outcomes of patients with acute respiratory distress syndrome (ARDS) by promoting the migration and regeneration of endogenous stem cells in the lungs. Furthermore, clinical data indicate that compared with traditional antibiotic treatment, MSC therapy can more effectively control infections and promote lung tissue repair (Esfandiary et al., 2024; Li et al., 2020). Optimal antibiotic concentration for pretreatment without reducing cell activity was determined by evaluating the survival rate of MSCS (viability). The effect of pretreatment on function of MSCs was evaluated by measuring the expression levels of antimicrobial peptide (AMP) genes (hepcidin and LL-37). However, the design and sample size of clinical trials still need to be further optimized to ensure the reliability and universality of the results.

### 4.3 The repair effect of MSCs on lung tissue injury

MSCs show promising prospects in repairing lung tissue damage. Studies have shown that MSCS can promote the regeneration and repair of lung tissue through multiple mechanisms, including secreting anti-inflammatory factors, regulating immune responses, and promoting cell regeneration. For instance, MSCs can significantly improve lung inflammation and damage caused by *Pseudomonas aeruginosa* infection by releasing growth factors and cytokines (Zhao et al., 2024). In addition, the research also found that MSCs can enhance the regeneration of alveolar epithelial cells and promote the recovery of functional lung tissue in acute lung injury models, thereby improving lung function (Doherty et al., 2023). These findings provide new ideas for the application of MSCs in the treatment of lung diseases. Future research should further explore the specific mechanism of action of MSCs and their application potential in clinical practice.

## 5 Mechanism exploration: the effect of MSCs on *Pseudomonas aeruginosa* infection

### 5.1 Cytokines and immune regulation

**Figure 1:**
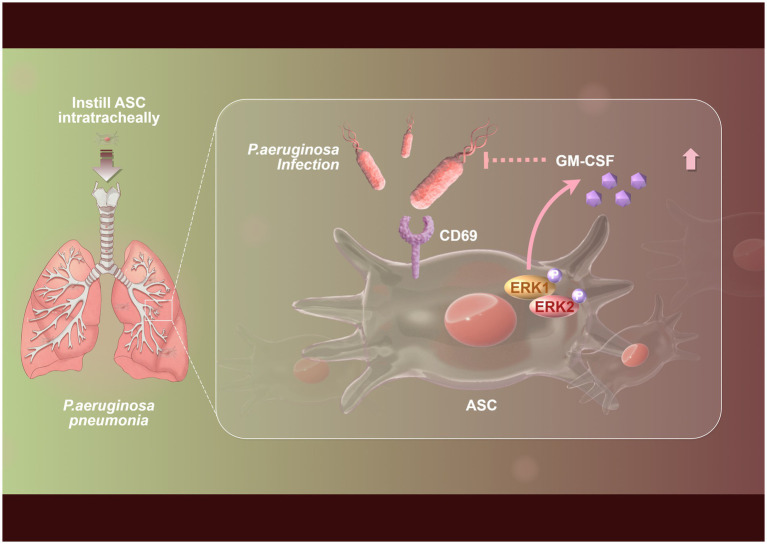
Flowchart of the mechanism by which mesenchymal stem cells inhibit *Pseudomonas aeruginosa* through ERK-GM-CSF immune signaling. The upregulation of CD69 expression in adipose tissue-derived mesenchymal stem cells (ASCs) was markedly enhanced during the infection by *Pseudomonas aeruginosa*, which subsequently enabled CD69 to recognize *Pseudomonas aeruginosa*. Following this recognition, the activation of the ERK1 pathway by CD69 led to increased secretion of granulocyte-macrophage colony-stimulating factor (GM-CSF) from ASCs, ultimately contributing to the mitigation of *Pseudomonas aeruginosa* pneumonia.

MSCs exert immunomodulatory effects by secreting various cytokines in response to *Pseudomonas aeruginosa* infection. For instance, studies have shown that adipose tissue-derived mesenchymal stem cells (ASCs) can recognize *Pseudomonas aeruginosa* through the C-type lectin receptor CD69 and regulate the secretion of granulocyte-macrophage colony-stimulating factor (GM-CSF) (Figure 1), thereby enhancing the body’s defense against infection (Jiang et al., 2021). In addition, MSCs can also inhibit inflammatory responses and alleviate tissue damage by secreting cytokines such as interleukins and tumor necrosis factors (Mitra et al., 2024). In models of acute lung infection, ASCs improve lung injury by inhibiting the activation of NLRC4 inflammasomes, reducing lung inflammation and bacterial load (Li et al., 2020). These studies demonstrate that MSCs enhance the host immune response and reduce *Pseudomonas aeruginosa* infections via cytokine regulation.

### 5.2 Apoptosis and regeneration

Apoptosis plays a significant role in the regeneration process mediated by MSCs. In the context of *Pseudomonas aeruginosa* infection, apoptosis is not only a necessary mechanism for eliminating damaged cells but also a signal for promoting regeneration. Studies have found that ASCs can upregulate circular GMP-AMP synthase (cGAS) after infection, activate the interferon (IFN) signaling pathway, and promote the secretion of IL-7. This process helps enhance the immune response of the body (Di et al., 2023). In addition, apoptotic signals participate in tissue remodeling and repair by promoting cell proliferation. The moderate occurrence of apoptosis can provide necessary cellular signals for regeneration, stimulate the proliferation and differentiation of surrounding cells, and further accelerate the tissue regeneration process (Vullien et al., 2021). Therefore, regulating the process of apoptosis is of great significance for enhancing the regeneration efficiency of MSCs after infection.

### 5.3 The influence of intercellular interactions

In the interaction between MSCs and *Pseudomonas aeruginosa* infection, the intercellular interaction is particularly important. Studies have shown that MSCs can establish complex signaling networks with surrounding immune cells and damaged tissue cells through direct intercellular contact and cytokines they secrete. The interaction between MSCs and macrophages can enhance the phagocytic ability of macrophages, thereby improving the clearance efficiency of *Pseudomonas aeruginosa*. In addition, by regulating intercellular signal transduction, MSCs can influence the functions of other immune cells and promote the enhancement of anti-infection immune responses. For instance, ASCs improves the phagocytic activity of macrophages and alleviates inflammatory damage in the lungs by inhibiting activation of NLRC4 inflammasomes (Li et al., 2020). These findings suggest MSCs can play multiple roles during infection through intercellular interactions, promoting the host’s immune defense and tissue repair.

### 5.4 The variability of MSC efficacy under different conditions

Under different conditions, when infected with *Pseudomonas aeruginosa*, therapeutic effect of MSCS shows significant variability, mainly affected by the following two factors:

#### 5.4.1 The pretreatment methods of MSC

Studies have shown that after MSC is pretreated with antibiotics (such as linezolid, etc.), the expression of its antimicrobial peptide genes (LL-37) is upregulated, thereby enhancing inhibitory and clearance ability against *Pseudomonas aeruginosa* (Esfandiary et al., 2024). However, antibacterial effect of untreated MSCS is relatively weak, indicating pretreatment conditions directly regulate the therapeutic effect of MSCS.

#### 5.4.2 Pathogenic factors of bacteria

The population sensing signaling molecules of *Pseudomonas aeruginosa* (such as OdDHL and HHQ) can interfere with the apoptotic pathway and cytokine secretion spectrum of MSCS. For instance, OdDHL and HHQ significantly promote the apoptosis of MSCS and induce the release of pro-inflammatory factors (IL-1β and IL-8), thereby weakening the survival rate and function of MSCS (Holban et al., 2014).

## 6 Future research directions and challenges

### 6.1 Standardization and optimization of MSCs treatment

Standardization and optimization are crucial for clinical transformation in stem cell therapy using MSCs, which face challenges like cell source heterogeneity and varying culture conditions that affect therapeutic consistency. Establishing strict production standards and quality control is essential, as cGMP-compliant MSCs production improves cell quality, safety, and therapeutic reproducibility (Sanz-Nogués and O'Brien, 2021). In addition, optimizing cell culture conditions, such as cell density, medium composition and temperature, can also significantly enhance the differentiation efficiency and biological activity of MSCs (Kubrova et al., 2019). Future research should focus on establishing a unified standardized operation process to ensure the quality and efficacy of MSCs, so as to promote their wide application in clinical practice.

### 6.2 Safety and effectiveness assessment

In the clinical application of MSCs therapy, the evaluation of safety and efficacy is an indispensable link. Although MSCs have shown good potential in regenerative medicine, their long-term safety still needs to be verified through rigorous clinical trials. Previous studies have pointed out that although MSCs have the ability of immune regulation and tissue repair, in some cases, there may be a risk of tumor occurrence. So, establishing a comprehensive evaluation system that covers the biological characteristics of cells, potential immune responses and clinical therapeutic effects is the foundation for ensuring the safe and effective application of MSCs. Furthermore, with the development of new biomarkers and imaging techniques, future research can utilize these tools for more precise efficacy monitoring and safety assessment, thereby providing stronger support for the clinical application of MSCs (Rebelatto et al., 2023).

Although MSCS have shown potential benefits in anti-inflammatory, immunomodulatory and tissue repair promotion in the treatment of pulmonary infections caused by *Pseudomonas aeruginosa*, their clinical application still faces multiple risks and limitations, which require careful evaluation. In terms of benefits, MSCS may improve the infection outcomes by down- regulating excessive inflammatory responses and enhancing host defense mechanisms; However, risks include the possibility of inconsistent efficacy due to the heterogeneity of MSC sources and batches, as well as the uncertainty of administration routes (such as intravenous injection which may trigger microembolism and procoagulant activity, while endotracheal administration may limit distribution) and timing (preventive use versus therapeutic application), which may affect safety and efficacy. Furthermore, immunosuppressive effect of MSCS may weaken the bacterial clearance ability and increase the risk of infection deterioration. Insufficient control of key quality attributes (such as cell activity and purity) during the manufacturing process may further amplify biosafety concerns. However, the differences between existing animal models (such as ARDS /LPS models) and real human *Pseudomonas aeruginosa* pneumonia limit the reliability of clinical translation. Therefore, future research needs to balance these factors under optimized dosage, standardized production and strict monitoring to ensure a risk–benefit balance and promote the safe transition of MSC therapy from experiments to clinical practice.

### 6.3 Combine the potential of other treatment methods

Combining other therapeutic methods to enhance the therapeutic effect of MSCs is one of the current research hotspots. Studies have shown that the combined application of MSCs with other treatment methods (such as gene therapy, drug therapy, etc.) can produce a synergistic effect and improve the overall therapeutic outcome. Furthermore, with the development of nanotechnology, the combination of drugs and MSCs using nanocarriers can achieve more precise drug delivery, thereby enhancing the targeting and effectiveness of treatment (Damiati et al., 2021). Besides, studies have shown that embedding MSCs in collagen-fibrin scaffolds can not only effectively inhibit *Pseudomonas aeruginosa* infection, but also promote the healing and regeneration of burn wounds (Mirshekar et al., 2023). Future research should continue to explore the combined use of MSCs with other therapeutic approaches, seeking the best combination strategy to achieve better therapeutic effects and patient prognosis.

### 6.4 Cost challenge: the cost of cell preparation is high

MSC treatment is not simply chemical drugs. It involves complex processes: donor screening, cell collection (from bone marrow or fat), *in vitro* amplification, purification, quality inspection (to ensure activity, purity and sterility), cryopreservation and transportation. Each step needs to be carried out under strict GMP (Good Manufacturing Practice) conditions, and the costs of facilities, reagents and labor are extremely high.

### 6.5 Scalability challenge: mass production bottleneck

It is relatively simple to prepare MSCS for a small number of patients in the laboratory, but it is a huge engineering challenge to stably produce sufficient doses of MSCS with uniform quality for tens of thousands of patients. It is necessary to establish a large-scale bioreactor cell culture system and ensure that the functional characteristics (immunomodulatory and antibacterial activity) of the cells do not change during the scale-up process.

### 6.6 Regulatory obstacles: complex approval process

MSC products fall under the category of “Advanced Therapeutic Medical Products (ATMP),” and their regulatory approval process is stricter and more complex than that of traditional drugs. Regulatory authorities require a large amount of evidence to prove its safety, effectiveness and quality controllability.

### 6.7 Drug treatment research on animal models of P*seudomonas pulmonary* infection

In addition to MSC treatment, research on drug therapy for animal models of *Pseudomonas pulmonary* infection has mainly focused on a natural product - garlic extract.

In animal model studies, garlic extract exerts therapeutic effects by inhibiting the Quorum Sensing (QS) system of bacteria. Quorum sensing is a key communication mechanism by which *Pseudomonas aeruginosa* regulates the expression of virulence factors, biofilm formation and antibiotic tolerance. The active ingredients in garlic can effectively block this system, making the originally protected biofilm bacteria vulnerable. The administration mechanism lies in the fact that when QS is inhibited, the tolerance of bacterial biofilms to antibiotics significantly decreases, and at the same time, their resistance to phagocytosis by host immune cells also declines. Furthermore, the research also found biofilms treated with garlic can more effectively activate the respiratory burst activity of PMNs, thereby enhancing the body’s innate immune clearance ability. In this study, a mouse lung infection model was used to evaluate therapeutic effect. The administration regimen was to orally administer garlic extract to mice for a total of 7 days, and prophylactic administration was initiated 2 days before infection. Although specific dosage of administration was not explicitly stated in the literature abstract, this study verified the feasibility of this administration method (Bjarnsholt et al., 2005).

## 7 Conclusion

In the research on treatment of *Pseudomonas aeruginosa* pulmonary infection, MSCs have demonstrated remarkable potential. In recent years, an increasing number of studies have shown that MSCs can not only alleviate inflammatory responses through their unique immunomodulatory properties, but also promote the repair and regeneration of lung tissue. These research advancements have provided new therapeutic ideas for severe pulmonary infections caused by *Pseudomonas aeruginosa*, potentially improving the prognosis and quality of life of patients.

However, in clinical applications, the therapeutic effect of MSC still faces many challenges. Firstly, the source, preparation method and characteristics of MSCS have a significant impact on the therapeutic effect. How to standardize these processes to ensure the consistency and safety of treatment is an urgent problem to be solved. In addition, factors such as the infusion method, dosage and treatment timing of MSCS also need further research to optimize the clinical plan. More importantly, the efficacy and safety of MSCS may vary among different individuals, which requires us to fully consider the individual differences of patients and the complexity of their conditions when conducting clinical trials.

Future research should focus on several key areas: First, to deeply explore the mechanism of action of MSCs in *Pseudomonas aeruginosa* infection to reveal its regulatory effect on the immune response; the second is to carry out large-scale clinical trials to verify the efficacy and safety of MSCs in infections at different stages. Thirdly, explore the combined application of MSC with other treatment methods, such as antibiotics or immunomodulators, to achieve a synergistic therapeutic effect. In addition, using genetic engineering technology to modify MSCs to make them more targeted and effective is also an important direction for future research.

MSCs have shown clinical translational potential in the treatment of *Pseudomonas aeruginosa* pulmonary infections, mainly based on their antibacterial and immunomodulatory properties. Research has found that MSCs can directly inhibit the growth of *Pseudomonas aeruginosa* and enhance the antibacterial effect by up-regulating antimicrobial peptide genes such as hepcidin and LL-37. Meanwhile, MSCs conditioned medium can accelerate epithelial repair and down-regulate inflammatory factors. However, the quorum sensing molecules of *Pseudomonas aeruginosa*, such as OdDHL, may interfere with the function of MSCs, induce apoptosis and alter the cytokine spectrum. This suggests that in clinical applications, antibiotic pretreatment or combination therapy should be combined to optimize the therapeutic effect. Overall, MSCs, as an adjuvant therapeutic approach, have the potential to promote infection control and tissue regeneration, but further research is needed to address the issue of bacterial interference.

In conclusion, MSCs have shown promising prospects in the treatment of pulmonary infections caused by *Pseudomonas aeruginosa*, but many challenges still need to be overcome in clinical application and research design. By integrating the research forces of multiple disciplines, more studies in the future are expected to promote the progress of this field and ultimately achieve more effective treatment for patients with lung infections.
