# Supplementary material for: Mesenchymal stromal cells for Pseudomonas aeruginosa pneumonia: mechanisms, preclinical evidence, and translational barriers
Source: Front Microbiol. 2025 Oct 20;16:1674456. doi: 10.3389/fmicb.2025.1674456 (PMC12580735; doi:10.3389/fmicb.2025.1674456)
Supplement: Supplementary file 1 [file Table_1.docx]

**Supplementary table** Treatment of *Pseudomonas aeruginosa* on lung Model with MSC

| **Aspect** | **Details** |
| --- | --- |
| Model | Cystic Fibrosis (CF) mouse model |
| Inoculum | Not specified |
| Strain | Not specified (clinical isolate, no specific designation provided) |
| Readouts | Bacterial burden (CFU), immune cell infiltration, pro-inflammatory cytokine levels (e.g., IL-6, TNF-α), survival rate, inflammatory damage (histology-related assessment, but specific methods not detailed) |
| MSC Source/Type | Mesenchymal stromal cell-derived extracellular vesicles (MSC EVs) |
| **Dose/Route** | **Dose**: Not specified (e.g., particle number or volume)  **Route**: Not specified (e.g., intratracheal or intravenous) |
| **Key Result** | 1. **Bacterial load**: Significantly reduced  2. **BAL cytokines**: Pro-inflammatory cytokine levels decreased  3. **Survival**: Survival rate improved  4. **Histology**: Inflammatory damage alleviated |
| **Limitations** | 1. Model details (inoculum, strain) not specified 2. Treatment parameters (MSC EV dose, administration route) not specified 3. All findings based on preclinical studies; no clinical data |
